# Comparative Genomic Analysis and Antimicrobial Resistance Profile of *Enterococcus* Strains Isolated from Raw Sheep Milk

**DOI:** 10.3390/vetsci12080685

**Published:** 2025-07-23

**Authors:** Anagnostou Glykeria-Myrto, Skarlatoudi Theodora, Theodorakis Vasileios, Bosnea Loulouda, Mataragas Marios

**Affiliations:** 1 Department of Dairy Research, Institute of Technology of Agricultural Products, Hellenic Agricultural Organization “DIMITRA”, Katsikas, 45221 Ioannina, Epirus, Greece; anmirto@yahoo.gr (A.G.-M.); theodoraskarlatoudi@hotmail.com (S.T.); bosnea@elgo.gr (B.L.); 2 General Agricultural Cooperative of Ioannina “Farmers’ Union”, Krya, 45500 Ioannina, Epirus, Greece; easikap@otenet.gr

**Keywords:** antimicrobial resistance, raw sheep milk, dairy farms, enterococci, bioinformatics, virulence

## Abstract

The role of *Enterococcus* spp. in food is debated since this group of lactic acid bacteria contains opportunistic pathogenic strains, some of which exhibit a multidrug-resistant profile. In livestock farms, the use of antibiotics is the most common practice to deal with mastitis-causing bacteria. However, the heavy usage and/or misuse of antibiotics has led to the emergence of antibiotic resistance. This study aimed to genetically and phenotypically characterize *Enterococcus* strains isolated from raw sheep milk. Samples were collected over one year from the bulk tank of a dairy sheep farm and cultured on selective media. Isolates were purified and analyzed by whole-genome sequencing and antimicrobial susceptibility testing. The isolates were divided into clusters and the corresponding species were identified along with their genes related to virulence and antibiotic resistance. The pan-, core- and accessory-genomes of the strains were determined. Finally, the antibiotic-resistant profile of selected strains was examined and associated with their genomic characterization. These findings contribute to a better understanding of *Enterococci* epidemiology, providing comprehensive profiles of their virulence and resistance genes. The presence of antibiotic-resistant bacteria in raw sheep milk destined for the production of cheese should raise awareness.

## Simple Summary

*Enterococcus* spp. are a type of bacteria that belong to the group called lactic acid bacteria, which are usually beneficial and often found in foods like dairy. But enterococci can be tricky—they can be both good and bad. They naturally live in the intestines of humans and animals or contribute to the formation of the unique flavor and texture of traditional raw milk cheeses. However, some enterococci species, especially *E. faecium* and *E. faecalis*, can cause serious infections in hospitals and are often resistant to many antibiotics. These bacteria are commonly found in raw sheep milk and cheese, and because they can acquire genes that make them resistant or harmful, they raise some concern—even though no outbreaks from food have been reported. This study looks at enterococci from Greek raw sheep milk to find out if they carry any harmful traits, using DNA sequencing and laboratory tests to assess their risk.

## 1. Introduction

*Enterococcus* spp. could be considered as a bad member of an otherwise good family (lactic acid bacteria—LAB). These microorganisms belong to a Gram-positive group of bacteria and can be found in a wide range of food and non-food commodities or niches. Enterococci have many faces, and we encounter them as a. commensal microorganisms in humans and animals that colonize the gastrointestinal tract, i.e., natural residents of the intestinal microflora, participating in the modulation of the immune system, and in several artisanal cheeses as part of the autochthonous bacterial community of the raw milk or processing environment, contributing to the organoleptic properties of the final products, b. probiotic microorganisms, e.g., *Enterococcus lactis* has been suggested as a probiotic feed supplement, c. an opportunistic pathogen-causing disease as strains of enterococci harbor virulence factors (VFs) and antimicrobial resistance genes (ARGs) that provide the ability to cause infection, a phenomenon highly relevant in nosocomial environments, and also to migrate from the digestive system to other tissues and organs [1,2].

Enterococci are highly related to dairy products impacting the processes of fermentation and the ripening of cheeses. They can be found as a standard part of the non-starter LAB (NSLAB) of many artisanal cheeses made from raw sheep, goat, or cow milk. They have a significant role during the fermentation of dairy products, contributing to the shaping of flavor, taste, and texture of these products. One third (1/3) and one fifth (1/5) of LAB isolated from cheeses and raw milk, respectively, are enterococci. The species that are most frequently isolated from raw milk and fermented dairy products are *E. faecium* and *E. faecalis*, but other species may also be isolated at a far lower frequency such as *E. hirae*, *E. italicus*, *E. durans*, *E. lactis*, *E. casseliflavus*, etc. [3,4,5].

The two species (*E. faecium* and *E. faecalis*) are related to enterococcal outbreaks in hospitals, with *E. faecalis* being the most commonly occurring species that cause nosocomial infections. The enterococcal genome is characterized by plasticity, meaning that enterococci can acquire VFs through horizontal gene transfer (HGT). Such VFs include virulent and resistance genes to different classes of antibiotics located on plasmids and/or mobile genetic elements (MGEs). For all these reasons, enterococci have not been granted the Generally Recognized As Safe (GRAS) label and therefore, have not been recommended for the Qualified Presumption of Safety (QPS) list proposed by the European Food Safety Authority (EFSA), although it should be mentioned that no enterococcal outbreak due to food consumption has been recorded so far [6,7,8].

*E. faecium* and *E. faecalis* are among the most antibiotic-resistant bacteria, and multidrug resistance (MDR), i.e., resistance to at least three different classes of antibiotics, contributes to their emergence as opportunistic pathogens. The antimicrobial resistance (AMR) displayed by enterococci represents a public health concern because of the potential human exposure through food consumption. The prevalence of ARGs in commensal enterococci may provide information on the selective pressure applied by the antibiotics on the intestinal microbial community in food-producing animals. AMR is one of the biggest global emergences, and these two enterococcal species are the most important active players because of their intrinsic or acquired resistance plus their dissemination of genetic determinants associated with resistance within and beyond the genus [7,9,10,11].

Consequently, the following research question was whether raw sheep milk could be a potential source and/or serve as a reservoir of antibiotic resistance and pathogenic traits of this commensal genus. The aim of this study was a. to study the enterococcal strains derived from raw sheep milk and enrich the presently insufficient data on the occurrence and VFs of enterococci derived from Greek raw sheep milk, which is widely used in cheese manufacturing, b. to identify the presence or not of the genetic elements related to virulence and resistance, employing not only genotypic techniques (whole-genome sequencing—WGS) but phenotypic methods (antimicrobial susceptibility testing—AST) as well.

## 2. Materials and Methods

### 2.1. Bacterial Strains

The dataset comprised 19 strains kept at −80 °C in De Man–Rogosa–Sharpe (MRS) broth (Condalab, Madrid, Spain, 1431), supplemented with 30% glycerol (Penta Chemicals, Prague, Czech Republic, 14530-11000PE) as a cryoprotectant agent. For LAB isolation from the raw sheep milk samples, two laboratory media were used: the MRS agar (Condalab, Madrid, Spain, 1433) and M17 agar (Condalab, Madrid, Spain, 1318). Incubation of the plates was conducted at 30 °C for 48–72 h (MRS), and at 22 °C and 37 °C for 48–72 h (M17). Before usage, the strains were revived twice in the appropriate broth medium for 24–48 h at 30 °C.

### 2.2. Genotyping—Whole-Genome Sequencing

Genomic DNA was isolated from enterococci strains and sequenced using Illumina short-reads technology (pair-end, 2 × 150 bp) [12]. The processing of the adapter-free raw fastq reads included quality control, polishing and de novo assembly into contigs, organization of the contigs into scaffolds, orientation of the scaffolds, quality assessment of the scaffolds (contamination and completeness), and evaluation of mis-assemblies after scaffolding. This analysis was performed as described in Apostolakos et al. (2023) [13].

The complete and draft genomes of the reference strains *E. faecalis* DSM20478 (complete), *E. hirae* FDAARGOS234 (complete), and *E. italicus* DRD111 (draft), acquired from the NCBI database (https://www.ncbi.nlm.nih.gov/, accessed on 5 June 2024), were used for scaffolding and orientation. The quality of the genome assemblies of enterococci was assessed with the CheckM v1.0.18 [14] and QUAST v5.2.0 [15] software, and only high-quality (completeness ≥ 95%, contamination ≤ 5%, heterogeneity 0%, number of scaffolds below 200, N50 > 30,000 bp, and genome size 2.80 Mbp ± 0.80 Mbp) draft genomes were retained.

### 2.3. Genotyping—Bioinformatic Analysis

Bioinformatic analysis was carried out using the Genome Taxonomy Database Toolkit (GTDB-Tk) [16], Type (Strain) Genome Server (TYGS) (https://tygs.dsmz.de/, accessed on 7 June 2024) [17], and Average Nucleotide Identity (ANI) values, calculated with the OrthANI software (https://www.ezbiocloud.net/, accessed on 7 June 2024) [18] for species identification; the PROKKA v1.14.5 [19] and eggnog-mapper v2.1.12 [20] for the annotation of enterococci genomes and the related functionality, respectively; the PathogenFinder v1.1 [21] for determining their pathogenic capacity on humans; the abricate v1.0.1 [22], amrfinderplus v3.11.26 [23], staramr 0.10.0 [24], and abriTAMR v1.0.15 [25] along with the reference databases VFDB v2.0 [26], MobileElementFinder v1.1.2 [27], ResFinder v4.7.2 [28], Antibiotic Resistance Gene Annotation (ARGANNOT) [29], Comprehensive Antibiotic Resistance Database (CARD) [30], Microbial Ecology Group Antimicrobial Resistances (MEGARes) [31], National Center for Biotechnology Information (NCBI) resistance gene [32], and PlasmidFinder v2.2 [33], for the identification of VFs, MGEs, ARGs, and plasmids.

MLST v2.0 [34] was employed for sequence type (ST) classification. Pangenome analysis and core genomic alignment were performed using the Roary v3.11.2 [35] tool. Proteins were classified into families based on amino acid sequence similarity (≥95%). If a gene appeared in at least 99% of isolates, it was included in the core genome. The phylogenetic relationships were determined using the kSNP v3.0 method [36] and the FastTree v2.1 program [37]. The resulting tree was built with the Interactive Tree of Life (iTOL) v6 [38].

All the aforementioned tools and software were run through the use of the integrated prokaryotic genome and pangenome analysis service (IPGA) v1.09 (https://nmdc.cn/ipga/, accessed on 7 June 2024) [39], the Center for Genomic Epidemiology (CGE) services (http://www.genomicepidemiology.org/services/, accessed on 7 June 2024), and the European public Galaxy server (https://usegalaxy.eu/, accessed on 7 June 2024) [40]. The hAMRonization tool [41] was applied to concatenate and summarize the results collected from the various bioinformatic techniques and methods. Finally, the traitar software (https://github.com/nick-youngblut/traitar3, accessed on 7 June 2024) was run to characterize the phenotypic traits of the enterococcal strains from their nucleotide or protein sequences [42].

The output file from Roary (a matrix denoting the presence and absence of genes within the enterococci genomes) was used to construct heatmaps and summary graphs with R software [43,44]. The BPGA v1.3 [45] program was used to visualize the distribution of the annotated Clusters of Orthologous Groups/Genes (COGs) and Kyoto Encyclopedia of Genes and Genomes (KEGG) categories. COG and KEGG heatmaps were created using the ImageGP webtool (https://www.bic.ac.cn/BIC/, accessed on 7 June 2024) [46]. All tools were run using the default parameters.

### 2.4. Phenotyping—Antimicrobial Susceptibility Testing

To support the genomic analysis of antimicrobial resistance genes in enterococci genomes, selected strains were tested for their susceptibility to various antimicrobial agents with the Sensititre™ MIC system (Thermo Fisher Scientific, Waltham, MA, USA), using the broth microdilution MIC method, according to the manufacturers’ instructions. The Sensititre™ EU Surveillance Enterococcus EUVENC AST Plate and Sensititre™ Mastitis CMV1AMAF Vet AST Plate from Thermo Fisher Scientific were used for performing the antimicrobial susceptibility testing (AST). The strains were classified as susceptible (S), resistant (R), or intermediate (I) using the European Committee on Antimicrobial Susceptibility Testing (EUCAST) Breakpoint Table v14.0 for *Enterococcus* spp. (https://www.eucast.org/clinical_breakpoints/, accessed on 28 February 2024) and Clinical and Laboratory Standards Institute (CLSI) Ed34 Breakpoints (https://clsi.org/resources/breakpoint-implementation-toolkit/, accessed on 28 February 2024).

## 3. Results and Discussion

### 3.1. Genome Assembly and Quality

In the current study, nineteen *Enterococcus* spp. strains derived from raw sheep milk were sequenced using short-reads technology (whole-genome sequencing—WGS). The assembly quality of the draft genomes regarding their completeness, contamination, heterogeneity, number of scaffolds, N50, and genome size is displayed in Table 1, while the genome properties are shown in Table 2. All genomes were of high quality and therefore were included in the downstream analysis.

The genome size of the enterococci isolates ranged from 2.74 to 2.97 Mbp (*E. faecalis* group, Table 3), from 2.22 to 2.48 Mbp (*E. italicus* group, Table 3), and 2.97 Mbp (*E. hirae* group, Table 3). Similarly, the GC content was from 37.3 to 37.6% (*E. faecalis* group), from 39.2 to 39.6% (*E. italicus* group), and 36.6% (*E. hirae* group). For all enterococci groups, the number of scaffolds varied between 1 and 180 with N50 to be within the range of 33,562 and 2,738,388 bp. The number of coding DNA sequences (CDS) and the number of genes found in enterococci were between 2173 and 2868, and between 2203 and 2930, respectively. Finally, the ribosomal RNAs (rRNA) found were from 2 to 7, the transfer RNAs (tRNA) were 26 to 58, and the transfer-messenger RNA (tmRNA) and repeat regions were 1 (Table 1 and Table 2).

### 3.2. Phylogenetic Relationships and Phenotypic Analysis

**Figure 1:**
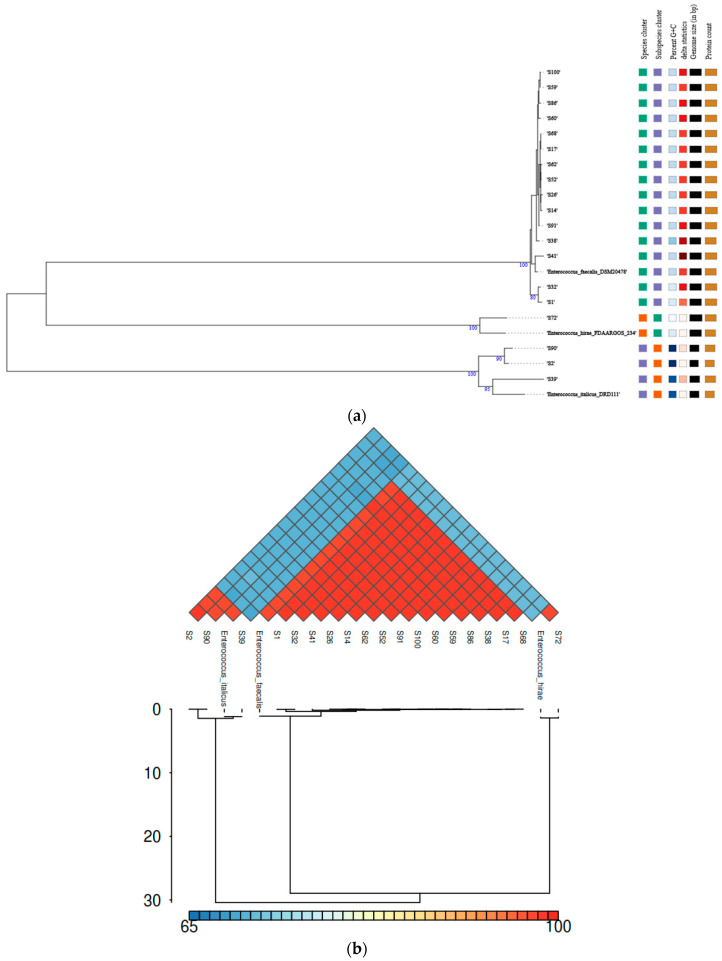
Genomic analysis of the enterococci isolates: (**a**) A TYGS-generated phylogram based on the proteome of the strains, including the reference *E. faecalis* DSM20478, *E. hirae* FDAARGOS234, and *E. italicus* DRD111 strains. (**b**) A combined heatmap–dendrogram graph representing the ANI values of the strains, including the reference *E. faecalis* DSM20478, *E. hirae* FDAARGOS234, and *E. italicus* DRD111 strains.

**Figure 2:**
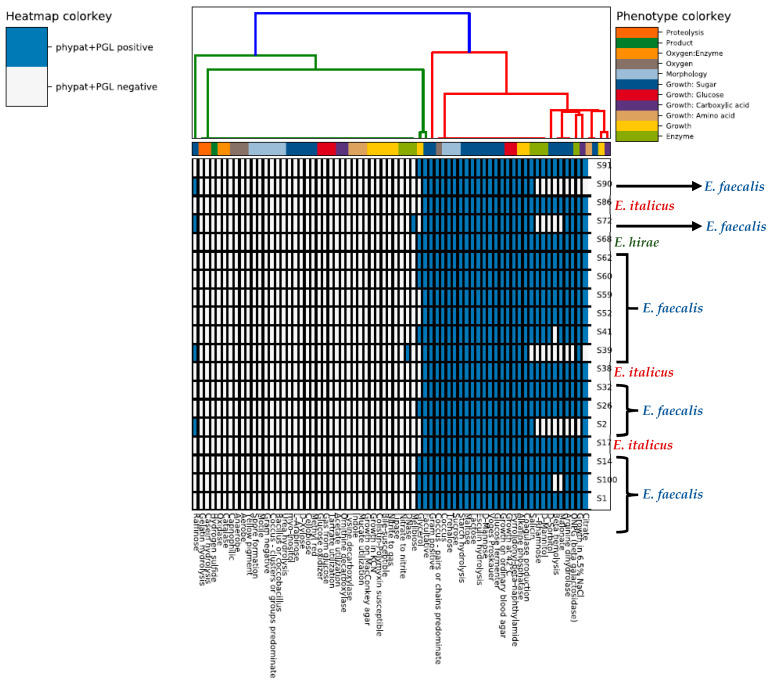
Heatmap depicting the phenotype properties of the nineteen whole-genome sequenced enterococci strains according to the WGS-based predictions made by the traitar program.

GTDB-Tk and TYGS (Figure 1a) assigned the strains in the following *Enterococcus* species: *E. faecalis* (78.95%), *E. italicus* (15.79%), and *E. hirae* (5.26%). ANI values verified the taxonomy of the isolates because the ANI value of each isolate, compared to its closest reference *Enterococcus* strain, exceeded the threshold (95–96%) for species-level delineation (Figure 1b). On the contrary, the strains that belonged to another species had an ANI value far below this limit. The species identification was further supported by the phylogenetic (Figure 1a) and digital DNA–DNA hybridization (dDDH) analyses. dDDH values varied from 99.0 to 99.7% and from 88.2 to 90.6% between the reference *E. faecalis* DSM20478 and *E. italicus* DRD111 strains, respectively, and the respective query isolates. The corresponding dDDH value between *E. hirae* FDAARGOS234 and S72 was 89.6%, indicating that all dDDH values were well above the suggested threshold (70%) for delineating species. Finally, the proteome-based phylogenetic tree (Figure 1a) shows different clusters which indicate that there are differences in the proteome between the strains (Figure 2).

The MLST typing of the isolates and the prediction of whether the strains are human pathogens or not are presented in Table 3, indicating the genetic diversity of the isolated enterococci. All *E. faecalis* isolates were recognized as human pathogenic strains with a high probability of being pathogens (87.4 to 88.8%). The other two species, *E. italicus* and *E. hirae,* were also identified as human pathogens (69.6 to 85.7%) although with a much lower probability for some isolates such as S2 (71.3%) and S72 (69.6%). Notably one isolation from the *E. italicus* group (S39) had a very low potential of being a human pathogen (39.4%). By observing the *E. italicus* group, consisting of three isolates, two strains were predicted as human pathogens (S2 and S90) and one strain (S39) as a non-pathogen. In addition, the two strains predicted as human pathogens had different probabilities, one strain with a high probability (S90, 85.7%) and one strain with a lower probability (S2, 71.3%) of being a human pathogen. This signifies the need for the systematic monitoring and genetic characterization of enterococci found in food products as there are significant differences in terms of their pathogenic potential even among strains of the same species. The analyzed draft genomes of the *E. faecalis* isolates were classified into two distinct sequence types (STs) (ST25 and ST326), with ST326 being the most prevalent (80%) ST.

To characterize the microbial strains from their sequences in terms of the phenotype traits we used the traitar tool (Figure 2) which confirmed the differences in the proteome between the different clusters/groups. The software predicted that all microorganisms were Gram-positive cocci able to hydrolyze esculin, a property that is commonly used in selective media to distinguish enterococci from other streptococci. The results revealed that the *E. faecalis* group was negative to raffinose (Raffinose^−^) but the other two groups of *E. italicus* and *E. hirae* were positive to this sugar (Raffinose^+^). On the other hand, all *E. italicus* and *E. hirae* strains were negative to the fermentation of the following sugars, L-rhamnose, D-mannitol, and D-sorbitol, but the opposite was true for *E. faecalis*. The only melibiose^+^ strain belonged to *E. hirae*. Independently from their taxonomy, all the isolated enterococci were lactose^+^ (a characteristic that indicates their adaptation to dairy environments) but only *E. faecalis* and *E. hirae* were ONPG^+^, i.e., *E. italicus* was ONPG^−^. The ONPG (ortho-Nitrophenyl-beta-D-galactopyranoside) test shows the presence or lack of the beta-galactosidase enzyme in a bacterial strain. This enzyme breaks down lactose into galactose and glycose and therefore, is an essential enzyme for lactose fermentation/metabolism. However, a strain negative to this test does not necessarily mean that this strain is not able to ferment lactose. Such microorganisms may use alternative pathways for lactose metabolism, e.g., the phosphoenolpyruvate (PEP)-dependent sugar-phosphotransferase system (PTS) and a cytoplasmic phospho-beta-galactosidase [47]. A noteworthy observation is the beta-hemolysis activity of *E. faecalis* (except for S41 and S100) which constitutes a virulence factor while the strains of *E. italicus* and *E. hirae* lack this enzyme.

Apart from the fermentation of various sugars, other important features for cheese manufacturing [48] include a. Starch hydrolysis: many bacteria can utilize starch as a carbon source for exopolysaccharide synthesis, which influences cheese texture. b. Acetoin and diacetyl production (positive to the Voges–Proskauer—VP test) of aroma-related compounds. c. Citrate metabolism (except of two strains from the *E. italicus* group, S39 and S90): many aromatic compounds found in cheese may originate from citrate as well. d. Growth at 42 °C (thermoduric). e. Growth in 6.5% NaCl (salt-tolerant), except of two strains from the *E. italicus* group (S2 and S90). These characteristics show the technological influence of enterococci, which are usually found in artisanal cheeses as part of the NSLAB, in the shaping of cheese sensory properties. Finally, *E. faecalis* and *E. hirae* were positive to arginine dihydrolase (arginine decarboxylase) which can result in the formation of putrescine (arginine decarboxylation to form agmatine and then agmatine deamination to produce putrescine), a biogenic amine that can lead to adverse health effects [48,49]. However, *E. italicus* lacked this feature.

### 3.3. Pangenome Analysis

**Figure 3:**
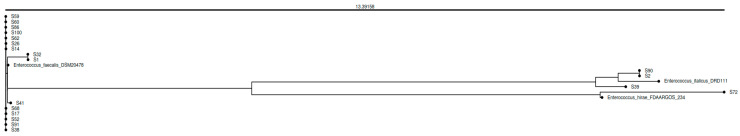
SNP-based phylogenetic analysis of the enterococci strains studied, including the reference *E. faecalis* DSM20478, *E. italicus* FDAARGOS234, and *E. hirae* DRD111 strains.

Phylogenetic analysis of the enterococci genomes was performed with the kSNP v3.0 software for building a single-nucleotide polymorphism (SNP)-based phylogenetic tree, which provides more discriminatory power. The analysis confirmed the existence of three different groups of species and some subgroups of strains within the species as follows: 1. *E. faecalis* group (15 strains) and two subgroups, each coincided with the respective ST: 1a. S14, S17, S26, S38, S52, S59, S60, S62, S68, S86, S91, and S100 (ST326) and 1b. S1, S32, and S41 (ST25); 2. *E. hirae* group (one strain, S72); 3. *E. italicus* (three strains) and two subgroups: 3a. S39 which was found to be a non-pathogenic strain and is separated from the other two strains, S2 and S90, which form the second (3b.) subgroup, supporting the genetic diversity results previously observed (Figure 3).

**Figure 4:**
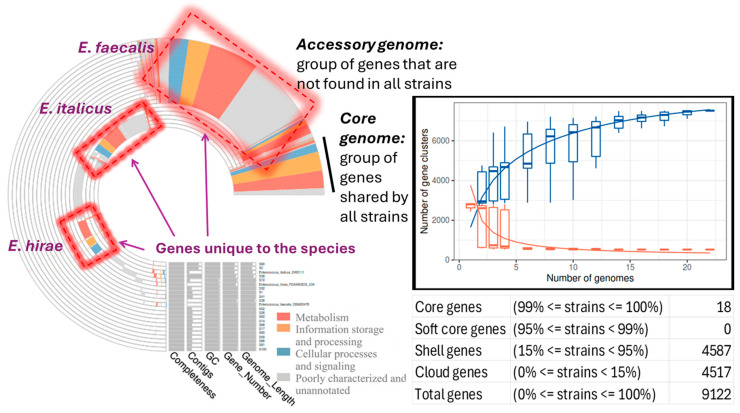
Pangenome analysis of the enterococci strains, including the reference *E. faecalis* DSM20478, *E. italicus* FDAARGOS234, and *E. hirae* DRD111 strains, showing the proportion of the core, shell, and cloud genes, the distribution of the core and accessory-unique genomes across the studied enterococci strains, the change in the size of the pan (blue boxes) and core (red boxes) genes as a function of the number of added sequenced genomes.

A pangenome analysis was carried out to show which genes are common and which are unique. The core genome is encompassed by only 18 genes present in at least 99% of the isolates (*n* = 23, including the reference strains) and represented 0.20% of the total number of genes (9122) (Figure 4). Therefore, the size of the accessory genome was extremely huge and almost exclusive (99.80%). It is separated into shell genes (15% ≤ genes present in strains < 95%; 4587 or 50.28%) and cloud or unique genes (0% ≤ genes present in strains < 15%; 4517 or 49.52%). Bacteria can horizontally transfer genes to other microbes via plasmids, and consequently their accessory genome can be large. This can significantly influence the size and content of the bacterial accessory genome since plasmids are MGEs that can carry genes for traits like AMR or virulence and therefore, bacterial accessory genomes often house genes for drug resistance and/or virulence [50,51,52,53].

The number of gene clusters present in the pangenome (core and accessory genome) increased with the number of genomes included in the analysis, whereas the number of gene clusters inside the core genome decreased reaching a plateau (Figure 4). This indicates that the number of new genes increases as new sequenced genomes are introduced into the pangenome, while the inclusion of new sequenced genomes does not substantially alter the core genome; this is an indication of the incremental genetic diversity that exists between the studied isolates (Figure 4).

**Figure 5:**
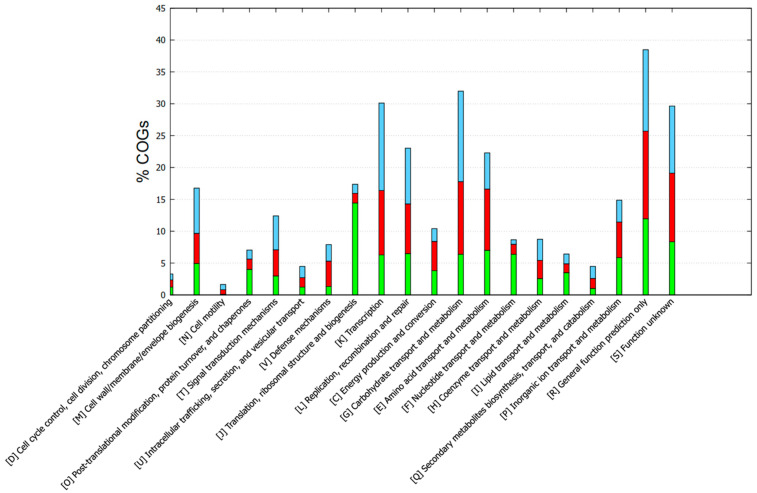
COG-based functional annotation of genes found in the core and accessory-unique genome of the enterococci strains. Green, core; red, accessory; cyan, unique.

**Figure 6:**
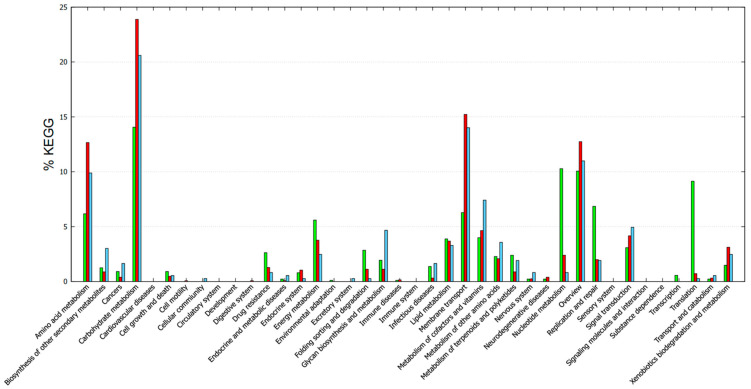
KEGG-based functional categories of genes found in the core and accessory-unique genome of the enterococci strains. Green, core; red, accessory; cyan, unique.

Functional annotation with GOG showed different categories/subsystems between the core and accessory genomes (Figure 5). The genes found in the core genome were mainly related to post-translational modification, protein turnover, and chaperones; translation; ribosomal structure and biogenesis; nucleotide transport and metabolism; and lipid transport and metabolism. The accessory-unique genome mainly included cell wall/membrane biogenesis, signal transduction mechanisms, defense mechanisms, transcription, replication, recombination, and repair. In addition, other mechanisms were also included such as energy production and conversion, the metabolism of carbohydrates, amino acids, and coenzymes (Figure 5). The KEGG functional annotation provided complementary information (Figure 6). Core genes were mostly associated with drug resistance, energy metabolism, folding, sorting and degradation, infectious diseases, lipid metabolism, nucleotide metabolism, replication and repair, transcription, and translation. Accessory-unique genes were assigned to functions associated with amino acid metabolism, carbohydrate metabolism, membrane transport, the metabolism of cofactors and vitamins, and signal transduction (Figure 6).

In essence, the functional categories assigned to the core genes are related to enterococcal survival, adaptation, and the ability to cause infections. On the other hand, accessory and unique genes that were linked to functions related to amino acid metabolism, carbohydrate metabolism, and membrane transport often play a role in acquiring and utilizing nutrients from diverse environments, contributing to the adaptability and survival of enterococci [54,55,56]. These properties are desirable in cheese manufacturing because they contribute to taste and flavor, making enterococci a significant focus in the study of these bacteria.

### 3.4. Antimicrobial Resistance

**Figure 7:**
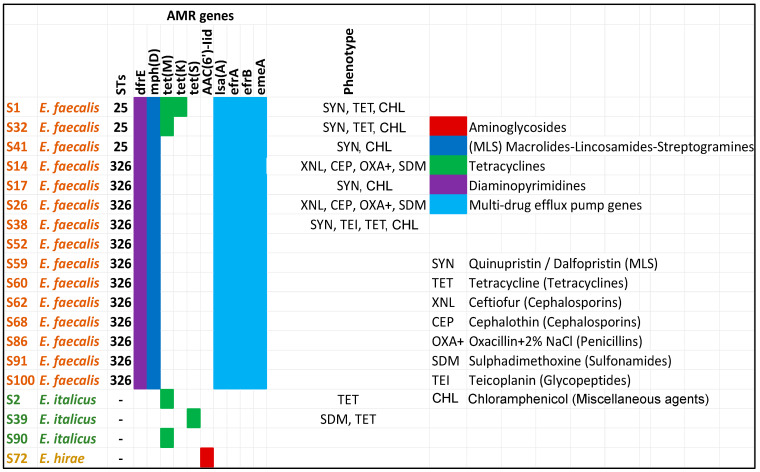
In silico screening of the whole-genome sequenced enterococci strains (draft genomes) for the identification of ARGs. Colors show the predicted phenotypes. “Phenotype” column refers to the AST results performed for only selected strains with the Sensititre^TM^ MIC platform. Symbols within the “phenotype” column indicate the antibiotic(s) to which a specific strain is resistant. Antibiotic symbols are explained on the right including the antibiotic class (inside the parenthesis) in which they belong. Strains S1, S2, S17, S32, S38, and S41 were tested with the Sensititre EU Surveillance Enterococcus EUVENC AST Plate whereas the strains S14, S26, and S39 were examined with the Sensititre VET Mastitis CMV1AMAF AST Plate.

Antimicrobial resistance, especially MDR, is an additional factor of virulence (pathogenicity). Therefore, the enterococci were screened for the presence of ARGs associated with different classes of antibiotics (Figure 7). The results showed that *E. faecalis*, compared with *E. italicus* and *E. hirae*, harbored an arsenal of genes related to AMR, from multi-drug efflux pump genes to genes conferring resistance to specific antibiotics.

In general, the AST results supported the in silico predictions. Some notable observations on the AMR of the tested strains are the following:

- All *E. faecalis* strains were resistant to Quinupristin/Dalfopristin—SYN (MLS—Macrolides–Lincosamides–Streptogramins) and Chloramphenicol. *E. faecalis* strains are intrinsically resistant to SYN. The S14 and S26 were not tested because a different AST plate was used which does not contain the SYN and CHL. Chloramphenicol is not considered in the breakpoints table of enterococci.
- Five out of the nine strains tested for resistance to Tetracyclines were found to be positive, i.e., they were resistant to TET. Decreased susceptibility to TET is frequent among enterococci [57,58].
- *E italicus* and *E. hirae* possessed genes conferring resistance to one antibiotic such as *tet* genes (*E. italicus*) and *aac*(*6*′)*-lid* (*E. hirae*). Indeed, both examined *E. italicus* strains (S2 and S39) showed resistance to tetracyclines.
- Among the tested strains, there were enterococci with resistance to three or more different classes of antibiotics such S1, S14, S26, S32, and S38. The last strain displayed resistance to four classes of antibiotics (MLS, Tetracyclines, Glycopeptides, and Chloramphenicol) whereas the other strains showed resistance to three classes (MLS, Tetracyclines, and Chloramphenicol or Cephalosporins, Penicillins, and Sulfonamides).
- All MDR strains belonged to the same group (*E. faecalis*). A high incidence of MDR *E. faecalis* strains has been observed in other studies as well [57]. The most prevalent MDR profile was the TET-ERY-CHL, i.e., antibiotics of the Tetracyclines, MLS, and Chloramphenicol classes, respectively, similar to the results obtained in the current work. Gião et al. (2022) [57] also found that *E. faecalis* strains were resistant to Chloramphenicol without detecting the *cfr* determinant in their genome, explaining that other phenicol resistance mechanisms are probably present.
- Resistance to ampicillin was not observed in any of the tested strains. This is in line with the observation that decreased susceptibility to this antibiotic is rare among *E. faecalis* strains [57,59]. A similar pattern (i.e., susceptibility) was obtained also for the Gentamycin.
- Enterococci showed some differences in their AMR profile, confirming the genetic diversity that exists among the isolated strains.
- The S38 (ST326) was a teicoplanin-resistant strain. The antibiotic belongs to the class of glycopeptides as vancomycin. The *vanA* gene is the most common cause of teicoplanin resistance in *E. faecalis*. The vanA genotype leads to resistance to both vancomycin and teicoplanin. The *vanZ* gene is also involved in teicoplanin resistance but not in vancomycin although its function is still unknown [60]. Interestingly, the S38 was the only strain among the tested strains that displayed a minimum inhibitory concentration (MIC) to vancomycin equal to 4 µg/mL which is the limit between a sensitive or resistant strain (S ≤ 4, R > 4). Nevertheless, no gene from the *van* operon was detected. The reason could be the inability of the software to detect the respective genes (i.e., fragmented genes). Vancomycin-resistant enterococci is a serious public health concern because the treatment of infections caused by these bacteria is challenging [61].
- All *E. faecalis* possessed multi-drug efflux pump genes. These systems have emerged as elements relevant to the intrinsic and acquired AMR of bacterial pathogens [60].
- Some strains (S14, S26, S38) showed resistance to antibiotics (XNL, CEP, and OXA+ for S14 and S26, and TEI and TET for S38) although no related genes were detected in their genomes. This, however, could be ascribed to other resistance and non-resistance factors like non-enzymatic mechanisms (e.g., the presence of efflux pump systems) or the presence of fragmented genes inside the draft genome of enterococci leading to low detection scores by the AMR databases, and thus, these genes remained unreported. Therefore, in silico screening for ARGs should always be accompanied by AST for reliable AMR determination, especially when ARG detection is based on draft genomes.

### 3.5. Virulence and Mobilome

An excessive variety of VFs were detected in all *E. faecalis* strains related to adherence, toxic effects, biofilm formation, etc. (Table 4). Notably, no VFs were found in the genome of the *E. italicus* and *E. hirae* groups.

Some genes related to biofilm formation and sex pheromones also contribute to the antimicrobial resistance of the microorganisms. Apart from the fact that a biofilm provides an environment of protection against the invasiveness of antibiotics or other antimicrobial agents, some genes involved in the biofilm formation could also affect the expression or activity of AMR-related genes. Sex pheromones may facilitate the transfer of genes associated with resistance through horizontal gene transfer (HGT). More specifically, in *E. faecalis*, genes associated with biofilm formation are also linked with antimicrobial resistance [62,63,64,65]. Pheromones secreted by *E. faecalis* can induce plasmid transfer carrying resistance genes [62,66,67].

Genes related to toxic effects were also found in *E. faecalis* strains. However, it should be mentioned that although the hyaluronidase enzyme (*hlyA*) was detected, which degrades hyaluronic acid (through the breakdown of connective tissue), the *cyl* genes encoding for cytolycins were not found, which are primarily associated with beta-hemolysis [68]. This is in contradiction to the previous in silico phenotypic analysis in which all *E. faecalis* strains (except for S41 and S100) were predicted as beta-hemolytic strains. The reason could be either due to an absence of the *cyl* genes or their presence as fragmented genes, which further supports the conclusions for AMR, i.e., in silico screening for pathogenic traits should be accompanied by laboratory tests for reliable determination, especially when draft genomes are used.

**Figure 8:**
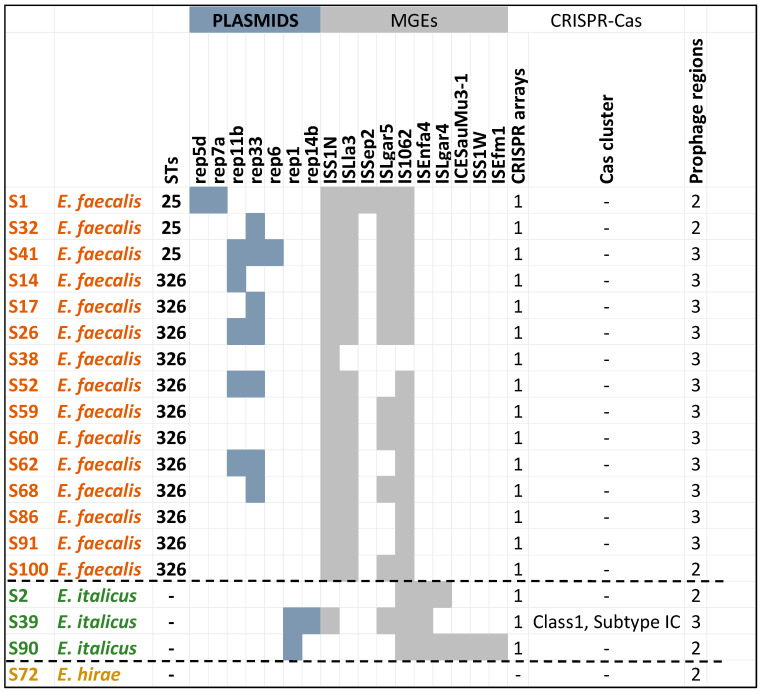
In silico screening of the whole-genome sequenced enterococci strains (draft genomes) for the identification of mobilome (collection of MGEs).

The mobilome of enterococci contained a diverse array of MGEs such as plasmids, insertion sequences, prophages, and clustered, regularly interspaced short palindromic repeats (CRISPR) arrays (Figure 8) which highlight their adaptability and ability to evolve in response to environmental pressures [69,70]. An interesting observation was that the content of the mobilome of *E. italicus* strains was different from those of *E. faecalis* while no MGEs were detected in *E. hirae* except two prophage regions. MGEs facilitate the acquisition and sharing of genetic material including genes related to virulence, adaptation, and antimicrobial resistance, enhancing enterococcal pathogenicity and making their resistance to macrolides, tetracyclines, quinolones, diaminopyrimidines, glycopeptides, and streptomycin increasingly common. Drug resistance through HGT mediated by MGEs is one of the main mechanisms that contribute to the spread of resistance genes between bacteria [1,70,71,72,73].

Another important finding worth mentioning was that the strains possessed orphan CRISPR loci (i.e., without Cas genes) in their genomes. CRISPR-Cas systems can limit the acquisition of MGEs, potentially influencing the evolution and adaptation of *E. faecalis* [74]. It has been suggested that orphan CRISPR arrays may be the leftovers of decaying CRISPR-Cas systems, or that the CRISPR system causes the loss of the Cas protein during the interaction with bacteria and foreign genes. The vast majority of orphan CRISPR are of unknown function. Studies have pointed out that the clinical isolates of MDR *E. faecalis* have more MGEs than and lack the complete CRISPR-Cas defense system of commensal enterococci. Previous works on *E. faecalis* draft genomes have indicated that there was a significant inverse correlation between the presence of CRISPR-Cas and acquired antibiotic resistance. In addition, orphan CRISPR-Cas can provide genomic defense in the presence of functional CRISPR-Cas encoding factors [75,76,77,78,79,80,81]. However, the S39 strains harbored a complete and intact CRISPR-Cas system. These systems provide protection against foreign genetic material [82,83]. Interestingly, this *E. italicus* strain was predicted as a non-pathogenic strain. The Cas cluster was of class 1 and subtype IC. Class 1 is known for its complex structure and multi-protein function including adaptation, expression, and interference [80,84,85,86,87,88,89].

## 4. Conclusions

This work’s objective was the investigation of whether enterococci could be a potential reservoir for antibiotic resistance and other pathogenic traits in livestock production systems, especially in raw sheep milk which is specifically used to produce various types of cheese in Greece. The results showed the high prevalence of clinically important species such as *E. faecalis*. To the best of our knowledge, no data has been reported until now about the presence of the specific *E. faecalis* STs (ST25 and ST326) found in raw sheep milk. Both STs were observed as emerging sequence types of multi-drug resistance. In particular, ST326 included an MDR *E. faecalis* strain with resistance to glycopeptides such as teicoplanin, which raises a public health concern as glycopeptides are very important antibiotics in human medicine. *E. faecalis* is a common resident of the gastrointestinal tract of farmed animals and its presence in raw sheep milk indicates that fecal contamination of raw milk has occurred.

Not all enterococci (*E. italicus* and *E. hirae*) isolated from raw sheep milk represented a substantial reservoir of virulence and antimicrobial resistance. However, the abundant species, i.e., *E. faecalis*, harbored unfavorable traits, including MDR, signifying their role as reservoirs for resistant genes and their subsequent transmission to other microorganisms. Also taking into consideration the fact that enterococci, including *E. faecalis*, are present in many types of cheese, the possibility of being a route for the transmission of antibiotic resistance should never be overlooked.

This study highlights several aspects of the virulence, pathogenicity, and antimicrobial resistance of enterococci isolated from raw sheep milk, aspects which are indications for their ongoing monitoring. This is also supported by the fact that some strains (S39) of the same species (*E. italicus*) were predicted as non-pathogenic while others (S2, S90) were predicted as pathogenic microorganisms—although no VFs were detected in all *E. italicus* and *E. hirae* isolates and additionally, the arsenal of their AMR genes was rather limited.

## Figures and Tables

**Table 1:** Genome assembly quality of the nineteen enterococci strains (draft genomes) and three reference strains.

Isolate ID	Completeness (%)	Contamination (%)	Heterogeneity (%)	No. of Scaffolds (≥300 bp)	N50 (bp) ^1^	Genome Size (bp)
S1	99.63	0.00	0	66	161,746	2,922,653
S2	99.01	0.00	0	140	33,562	2,220,060
S14	99.63	0.19	0	70	194,382	2,968,082
S17	99.25	0.19	0	67	194,381	2,926,349
S26	99.63	0.19	0	68	151,115	2,949,559
S32	99.63	0.19	0	56	160,970	2,930,115
S38	99.63	0.00	0	1	2,738,388	2,738,388
S39	99.01	0.00	0	180	48,375	2,480,341
S41	99.63	0.19	0	69	140,911	2,887,362
S52	99.63	0.19	0	60	194,228	2,942,042
S59	99.63	0.00	0	59	194,228	2,924,412
S60	99.63	0.00	0	61	194,228	2,924,361
S62	99.63	0.19	0	63	194,228	2,959,622
S68	99.25	0.19	0	60	194,227	2,919,192
S72	99.63	0.00	0	37	217,569	2,972,779
S86	99.63	0.00	0	62	194,228	2,934,362
S90	99.01	0.00	0	142	37,001	2,333,246
S91	99.63	0.00	0	60	194,228	2,906,786
S100	99.63	0.00	0	58	194,228	2,869,983
Ref. Ef ^2^	99.27	2.21	0	5	3,005,854	3,245,651
Ref. Eh ^2^	98.84	0.17	0	1	2,845,651	2,845,651
Ref. Ei ^2^	97.45	2.01	0	28	86,107	2,334,547

^1^ The sum of the lengths of all contigs of size N50 or longer contain at least 50% of the total genome sequence. ^2^ Reference strains (complete genomes except of *E. italicus* DRD111): Ef, *E. faecalis* DSM20478, one chromosome and four plasmids; Eh, *E. hirae* FDAARGOS234; and Ei, *E. italicus* DRD111.

**Table 2:** Genome properties of the nineteen enterococci strains (draft genomes) and three reference strains.

Isolate ID ^1^	No. of CDSs ^2^	No. of Genes	GC Content (%)	Repeat Region	rRNA ^2^	tRNA ^2^	tmRNA ^2^
S1	2806	2864	37.3	1	3	54	1
S2	2173	2203	39.6	1	3	26	1
S14	2868	2930	37.4	1	3	58	1
S17	2835	2896	37.4	1	3	57	1
S26	2856	2918	37.4	1	3	58	1
S32	2819	2878	37.3	1	3	55	1
S38	2624	2685	37.6	1	6	54	1
S39	2450	2516	39.2	1	7	58	1
S41	2800	2857	37.5	1	4	52	1
S52	2847	2904	37.4	1	3	53	1
S59	2817	2874	37.5	1	3	53	1
S60	2815	2872	37.5	1	3	53	1
S62	2858	2915	37.4	1	3	53	1
S68	2829	2886	37.4	1	3	53	1
S72	2649	2710	36.6	1	3	57	1
S86	2819	2876	37.5	1	3	53	1
S90	2280	2322	39.4	1	2	39	1
S91	2805	2862	37.5	1	3	53	1
S100	2740	2797	37.5	1	3	53	1
Ref. Ef ^2^	2754	2830	37.5	1	12	63	1
Ref. Eh ^2^	2483	2570	37.0	2	18	68	1
Ref. Ei ^2^	2240	2281	39.0	2	2	38	1

^1^ Reference strains (complete genomes except of *E. italicus* DRD111): Ef, *E. faecalis* DSM20478, one chromosome and four plasmids; Eh, *E. hirae* FDAARGOS234; and Ei, *E. italicus* DRD111. ^2^ CDSs, Coding DNA Sequences; rRNA, ribosomal RNA; tRNA, transfer RNA; tmRNA, transfer-messenger RNA. Not found by the PROKKA annotation tool.

**Table 3:** MLST typing and human pathogen prediction of the nineteen enterococci strains (draft genomes).

Isolate ID	Taxonomy	MLST	Human Pathogen
S1	*E. faecalis*	ST25	Yes (0.887) ^1^
S14	*E. faecalis*	ST326	Yes (0.875)
S17	*E. faecalis*	ST326	Yes (0.874)
S26	*E. faecalis*	ST326	Yes (0.874)
S32	*E. faecalis*	ST25	Yes (0.873)
S38	*E. faecalis*	ST326	Yes (0.888)
S41	*E. faecalis*	ST25	Yes (0.881)
S52	*E. faecalis*	ST326	Yes (0.875)
S59	*E. faecalis*	ST326	Yes (0.888)
S60	*E. faecalis*	ST326	Yes (0.888)
S62	*E. faecalis*	ST326	Yes (0.875)
S68	*E. faecalis*	ST326	Yes (0.874)
S86	*E. faecalis*	ST326	Yes (0.884)
S91	*E. faecalis*	ST326	Yes (0.888)
S100	*E. faecalis*	ST326	Yes (0.888)
S2	*E. italicus*	- ^2^	Yes (0.713)
S39	*E. italicus*	- ^2^	No (0.394)
S90	*E. italicus*	- ^2^	Yes (0.857)
S72	*E. hirae*	- ^2^	Yes (0.696)

^1^ Whether the microorganism is predicted as human pathogenic (yes or no) and the probability of being human pathogenic (inside the parenthesis). ^2^ MLST profile is not available for this species.

**Table 4:** VFs found in the whole-genome sequenced *E. faecalis* strains (draft genomes).

Isolate ID ^1^	Biofilm	Adherence	Exoenzyme	Capsule	Sex Pheromones	Antiphagocytic Activity	Oxidative and Thermal Resistance
S1	*ebpABC*, *srtA*, *srtC* (*bps*), *bopD*, *fsrC*	*efaAfs*, *fss1*, *fss3*	*gelE*, *hylA*, *sprE*	*cpsA*, *cpsB*	*cCF10*, *cOB1*, *cad*, *camE*	*elrA*	*tpx*, *clpC*
S32	*ebpABC*, *srtA*, *srtC* (*bps*), *bopD*, *fsrC*	*ace*, *efaAfs*, *fss1*, *fss3*	*gelE*, *hylA*, *sprE*	*cpsA*, *cpsB*	*cCF10*, *cOB1*, *cad*, *camE*	*elrA*	*tpx*, *clpC*
S41	*ebpABC*, *srtA*, *srtC* (*bps*), *bopD*, *fsrC*	*ace*, *efaAfs*, *fss1*, *fss3*	*gelE*, *hylA*, *sprE*	*cpsA*, *cpsB*	*cCF10*, *cOB1*, *cad*, *camE*	*elrA*	*tpx*, *clpC*
S14	*ebpABC*, *srtA*, *srtC* (*bps*), *bopD*, *fsrC*	*ace*, *efaAfs*, *fss1*, *fss3*	*gelE*, *hylA*, *sprE*	*cpsA*, *cpsB*	*cCF10*, *cOB1*, *cad*, *camE*	*elrA*	*tpx*, *clpC*
S17	*ebpABC*, *srtA*, *srtC* (*bps*), *bopD*, *fsrC*	*ace*, *efaAfs*, *fss1*, *fss3*	*gelE*, *hylA*, *sprE*	*cpsA*, *cpsB*	*cCF10*, *cOB1*, *cad*, *camE*	*elrA*	*tpx*, *clpC*
S26	*ebpABC*, *srtA*, *srtC* (*bps*), *bopD*, *fsrC*	*ace*, *efaAfs*, *fss1*, *fss3*	*gelE*, *hylA*, *sprE*	*cpsA*, *cpsB*	*cCF10*, *cOB1*, *cad*, *camE*	*elrA*	*tpx*, *clpC*
S38	*ebpABC*, *srtA*, *srtC* (*bps*), *bopD*, *fsrC*	*ace*, *efaAfs*, *fss1*, *fss3*	*gelE*, *hylA*, *sprE*	*cpsA*, *cpsB*	*cCF10*, *cOB1*, *cad*, *camE*	*elrA*	*tpx*, *clpC*
S52	*ebpABC*, *srtA*, *srtC* (*bps*), *bopD*, *fsrC*	*ace*, *efaAfs*, *fss1*, *fss3*	*gelE*, *hylA*, *sprE*	*cpsA*, *cpsB*	*cCF10*, *cOB1*, *cad*, *camE*	*elrA*	*tpx*, *clpC*
S59	*ebpABC*, *srtA*, *srtC* (*bps*), *bopD*, *fsrC*	*ace*, *efaAfs*, *fss1*, *fss3*	*gelE*, *hylA*, *sprE*		*cCF10*, *cOB1*, *cad*, *camE*	*elrA*	*tpx*, *clpC*
S60	*ebpABC*, *srtA*, *srtC* (*bps*), *bopD*, *fsrC*	*ace*, *efaAfs*, *fss1*, *fss3*	*gelE*, *hylA*, *sprE*		*cCF10*, *cOB1*, *cad*, *camE*	*elrA*	*tpx*, *clpC*
S62	*ebpABC*, *srtA*, *srtC* (*bps*), *bopD*, *fsrC*	*ace*, *efaAfs*, *fss1*, *fss3*	*gelE*, *hylA*, *sprE*		*cCF10*, *cOB1*, *cad*, *camE*	*elrA*	*tpx*, *clpC*
S68	*ebpABC*, *srtA*, *srtC* (*bps*), *bopD*, *fsrC*	*ace*, *efaAfs*, *fss1*, *fss3*	*gelE*, *hylA*, *sprE*		*cCF10*, *cOB1*, *cad*, *camE*	*elrA*	*tpx*, *clpC*
S86	*ebpABC*, *srtA*, *srtC* (*bps*), *bopD*, *fsrC*	*ace*, *efaAfs*, *fss1*, *fss3*	*gelE*, *hylA*, *sprE*		*cCF10*, *cOB1*, *cad*, *camE*	*elrA*	*tpx*, *clpC*
S91	*ebpABC*, *srtA*, *srtC* (*bps*), *bopD*, *fsrC*	*ace*, *efaAfs*, *fss1*, *fss3*	*gelE*, *hylA*, *sprE*		*cCF10*, *cOB1*, *cad*, *camE*	*elrA*	*tpx*, *clpC*
S100	*ebpABC*, *srtA*, *srtC* (*bps*), *bopD*, *fsrC*	*ace*, *efaAfs*, *fss1*, *fss3*	*gelE*, *hylA*, *sprE*		*cCF10*, *cOB1*, *cad*, *camE*	*elrA*	*tpx*, *clpC*

^1^ S1, S32, and S41 belong to ST25; the other strains belong to ST326.

## Data Availability

The whole-genome sequencing data have been deposited at GenBank (NCBI) under accession (BioProject) number PRJNA1294297 (https://www.ncbi.nlm.nih.gov/).

## Abbreviations

The following abbreviations are used in this manuscript:

VFs	Virulence Factors
AMR	Antimicrobial Resistance
MGEs	Mobile Genetic Elements
MRS	De Man–Rogosa–Sharpe
EUCAST	European Committee on Antimicrobial Susceptibility Testing
CLSI	Clinical and Laboratory Standards Institute
AST	Antimicrobial Susceptibility Testing
MDR	Multidrug Resistant
ANI	Average Nucleotide Identity
MLST	Multi-Locus Sequence Typing
CGE	Center for Genomic Epidemiology
iTOL	Interactive Tree of Life
ARGANNOT	Antibiotic Resistance Gene Annotation
CARD	Comprehensive Antibiotic Resistance Database
NCBI	National Center for Biotechnology Information
TYGS	Type (Strain) Genome Server
GTDB-Tk	Genome Taxonomy Database Toolkit
MEGARes	Microbial Ecology Group Antimicrobial Resistances
ARGs	Antimicrobial Resistance Genes
WGS	Whole-Genome Sequencing
CDS	Coding DNA Sequence
rRNA	Ribosomal RNA
tRNA	Transfer RNA
tmRNA	Transfer-Messenger RNA
dDDH	Digital DNA–DNA Hybridization
COGs	Clusters of Orthologous Groups/Genes
SNPs	Single Nucleotide Polymorphisms
KEGG	Kyoto Encyclopedia of Genes and Genomes
ONPG	Ortho-Nitrophenyl-beta-D-galactopyranoside
PEP	Phosphoenolpyruvate
PTS	Phosphotransferase system
MLS	Macrolides–Lincosamides–Streptogramins
MIC	Minimum Inhibitory Concentration
HGT	Horizontal Gene Transfer
CRISPR	Clustered, Regularly Interspaced Short Palindromic Repeats
Cas	CRISPR-associated gene
